# Supplementary material for: PTPRN2 and PLCβ1 promote metastatic breast cancer cell migration through PI(4,5)P2‐dependent actin remodeling
Source: EMBO J. 2015 Nov 30;35(1):62–76. doi: 10.15252/embj.201591973 (PMC4717998; doi:10.15252/embj.201591973)
Supplement: Supplementary file 1 — Appendix [file EMBJ-35-062-s001.pdf]

APPENDIX

**Appendix Figures**	**Page 2**
**Appendix Figure Legends**	**Page 9**
**Appendix Tables**	**Page 13**
**Appendix Supplementary Methods**	**Page 15**
**Appendix References**	**Page 17**

Appendix Figure 1

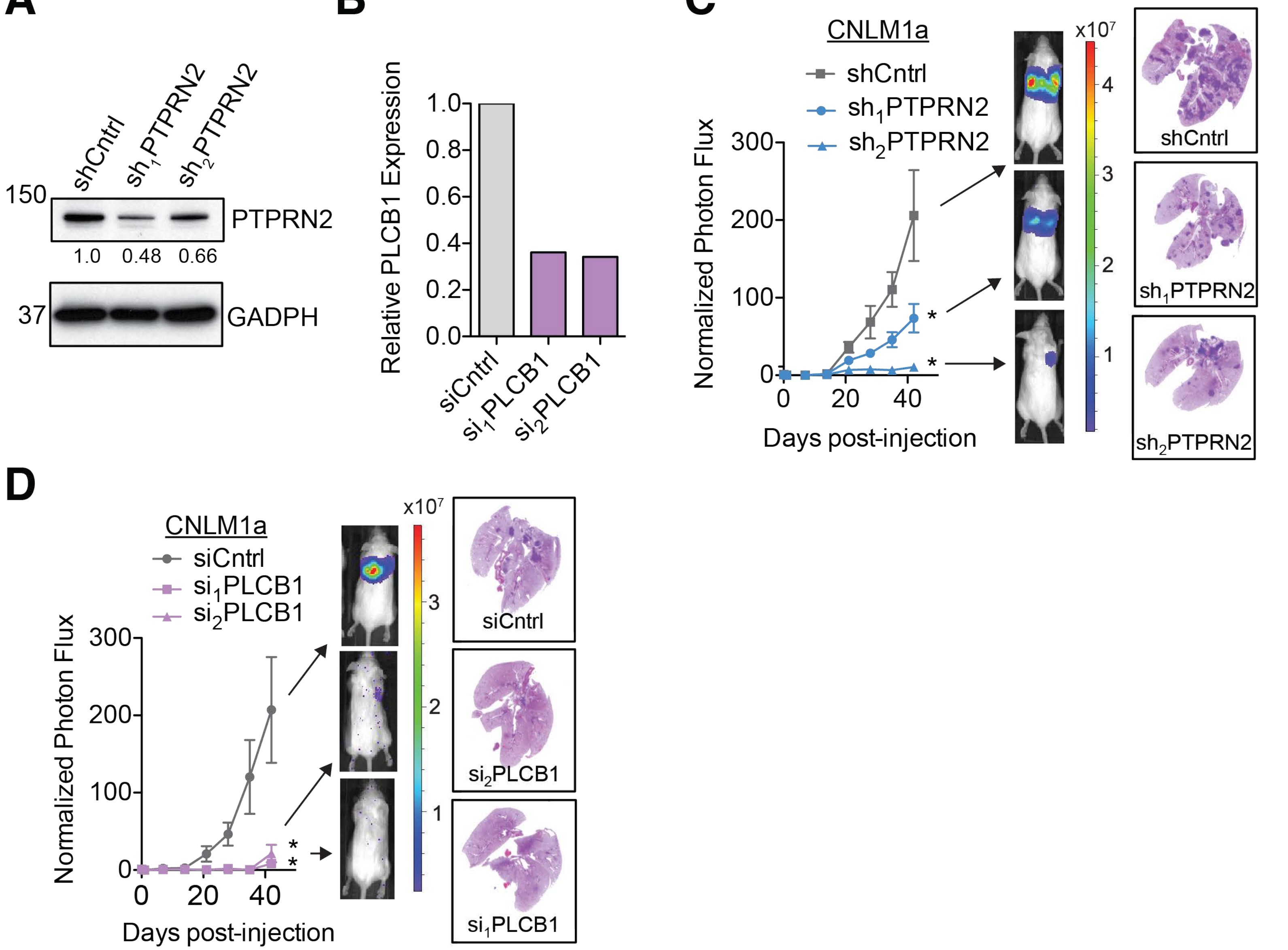

Appendix Figure 2

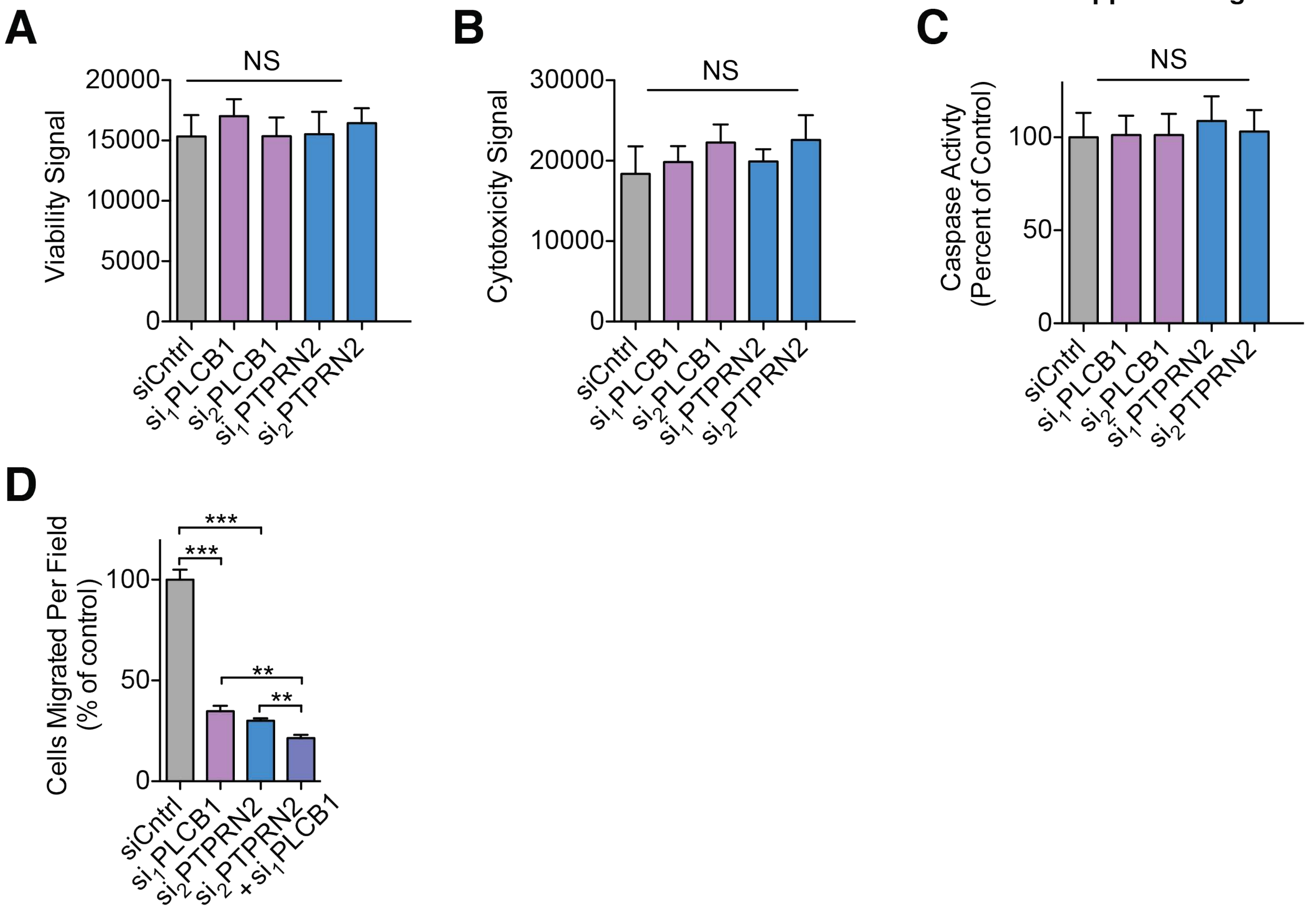

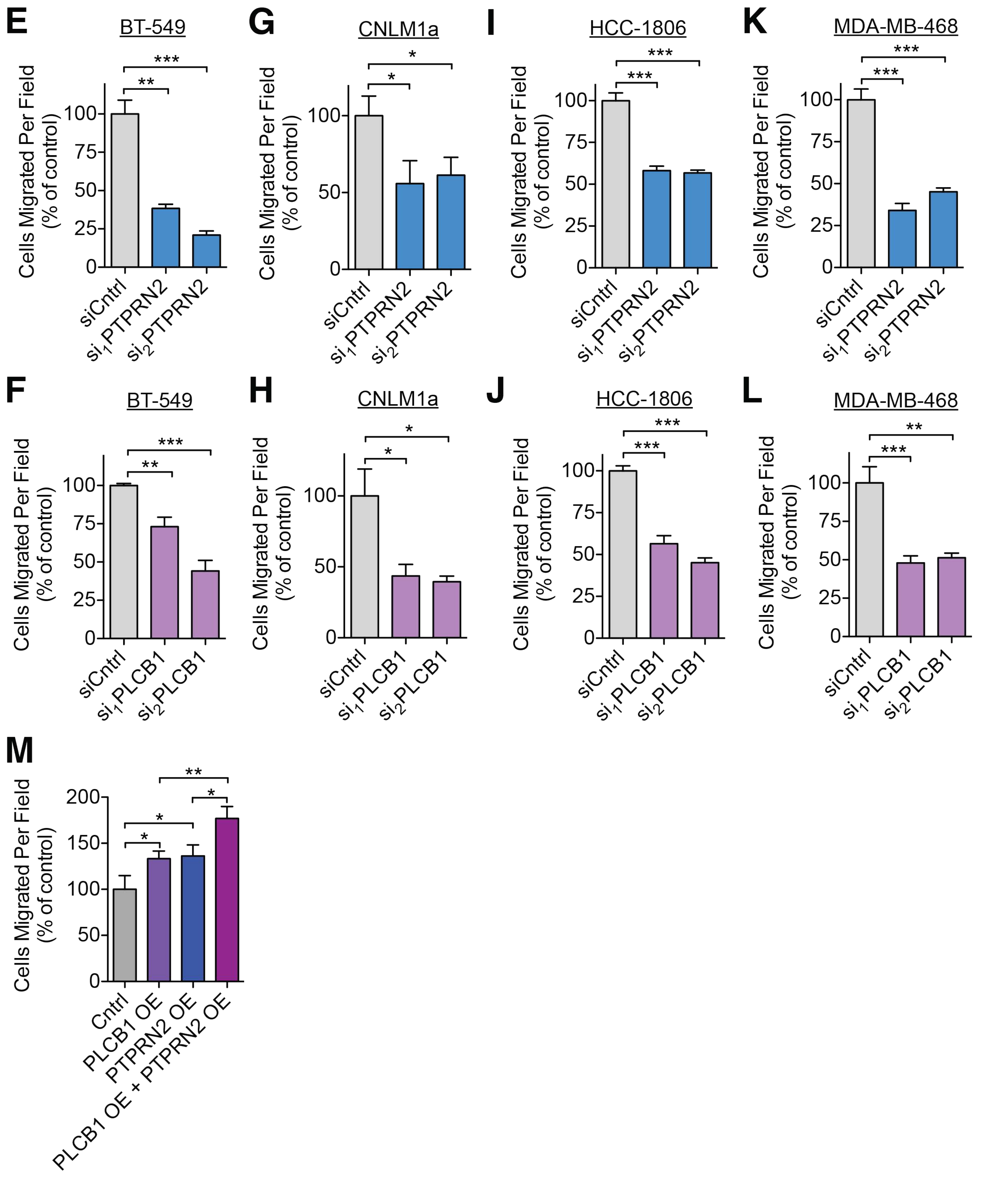
E
BT-549
G
CNLM1a
I
HCC-1806
K
MDA-MB-468
Cells Migrated Per Field (% of control)
siCntrl
si1PTPRN2
si2PTPRN2
F
BT-549
H
CNLM1a
J
HCC-1806
L
MDA-MB-468
si1PLCB1
si2PLCB1
M
Cntrl
PLCB1 OE
PTPRN2 OE
PLCB1 OE + PTPRN2 OE

Appendix Figure 3

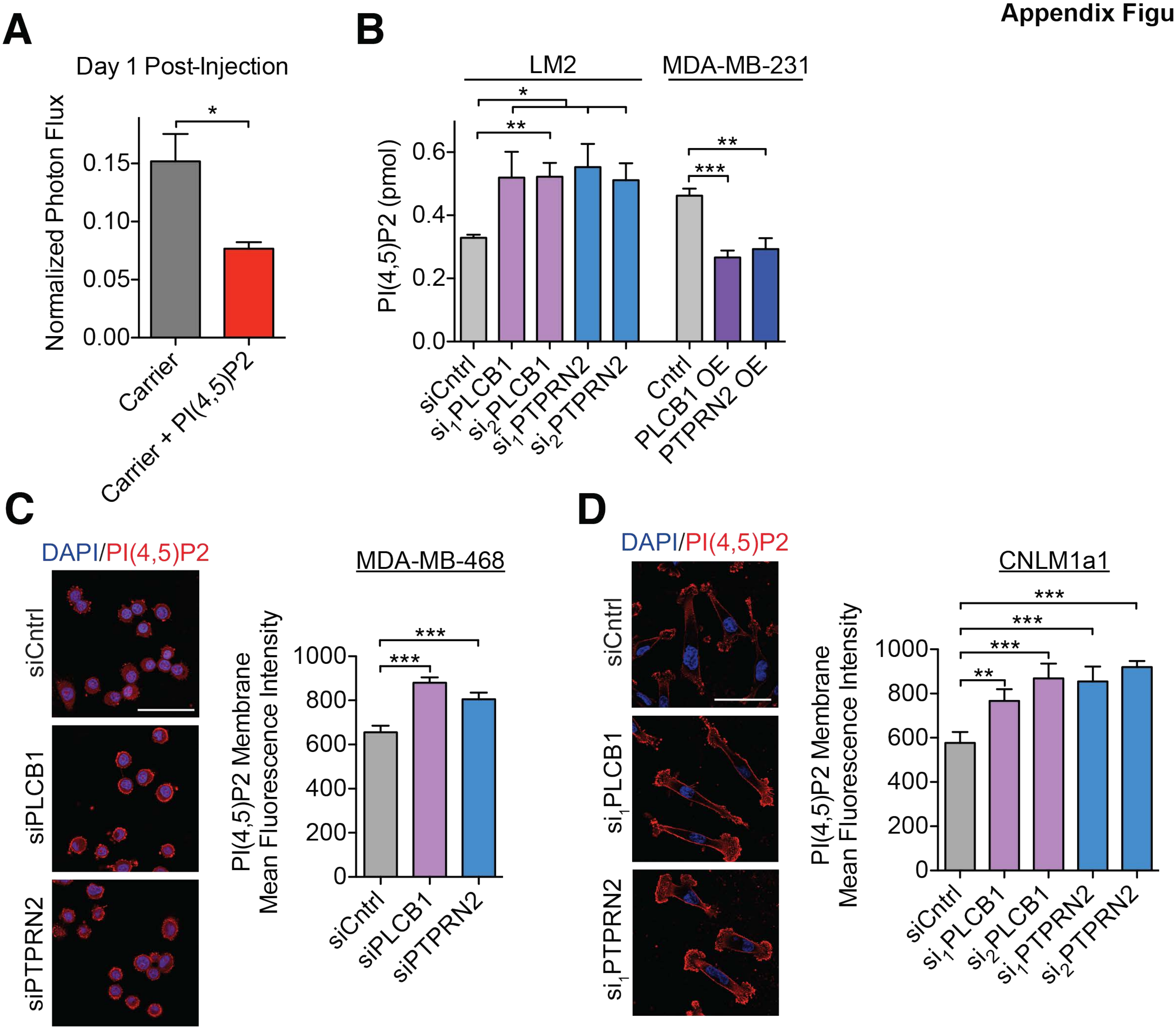

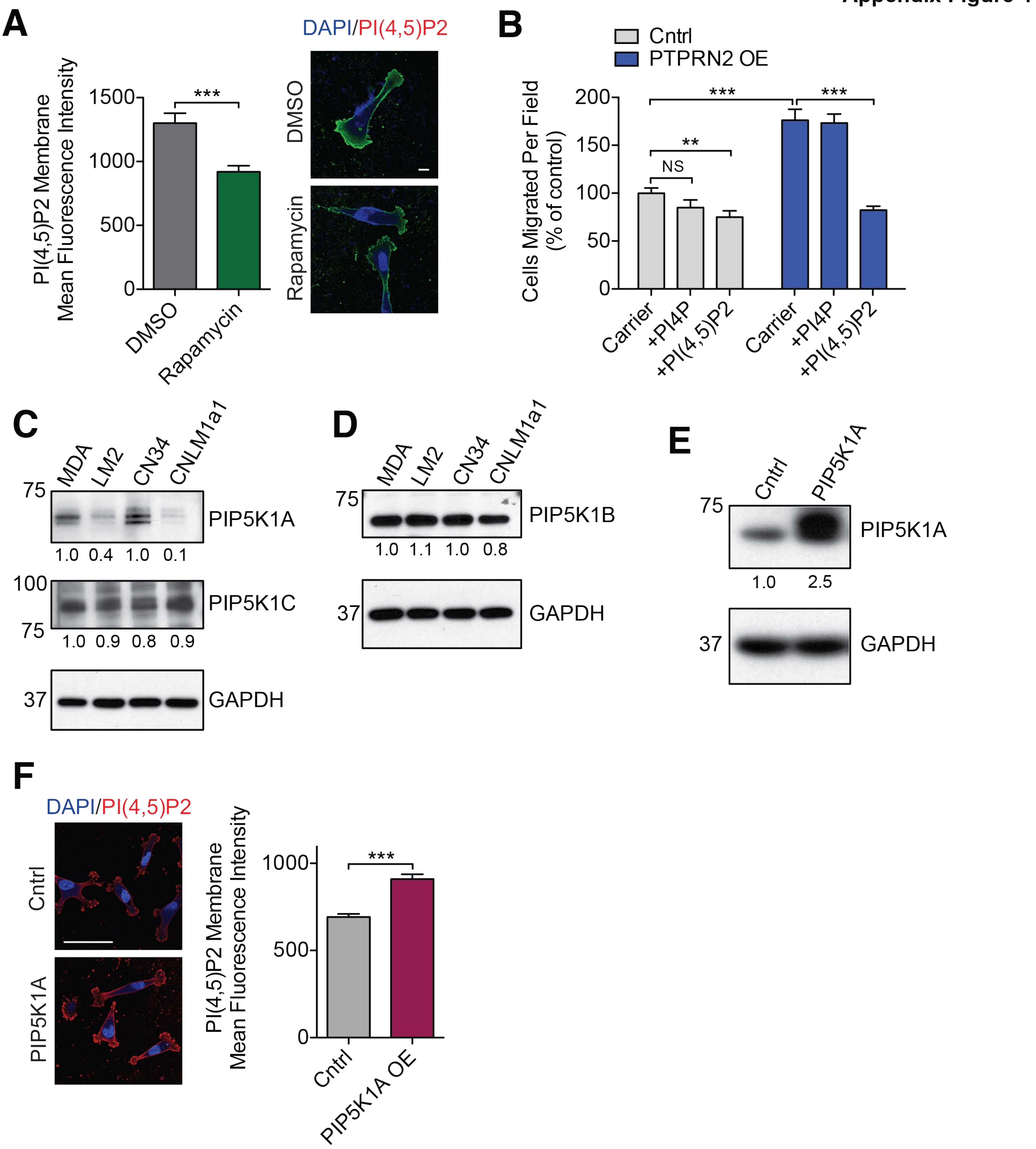
A
DAPI/PI(4,5)P2
PI(4,5)P2 Membrane Mean Fluorescence Intensity
1500
1000
500
0
***
DMSO
Rapamycin
DMSO
Rapamycin
B
Cntrl
PTPRN2 OE
Cells Migrated Per Field (% of control)
200
150
100
50
0
***
***
**
NS
Carrier
+PI4P
+PI(4,5)P2
Carrier
+PI4P
+PI(4,5)P2
C
MDA
LM2
CN34
CNLM1a1
75
PIP5K1A
1.0 0.4 1.0 0.1
100
75
PIP5K1C
1.0 0.9 0.8 0.9
37
GAPDH
D
MDA
LM2
CN34
CNLM1a1
75
PIP5K1B
1.0 1.1 1.0 0.8
37
GAPDH
E
Cntrl
PIP5K1A
75
PIP5K1A
1.0 2.5
37
GAPDH
F
DAPI/PI(4,5)P2
Cntrl
PIP5K1A
PI(4,5)P2 Membrane Mean Fluorescence Intensity
1000
500
0
***
Cntrl
PIP5K1A OE

Appendix Figure 5

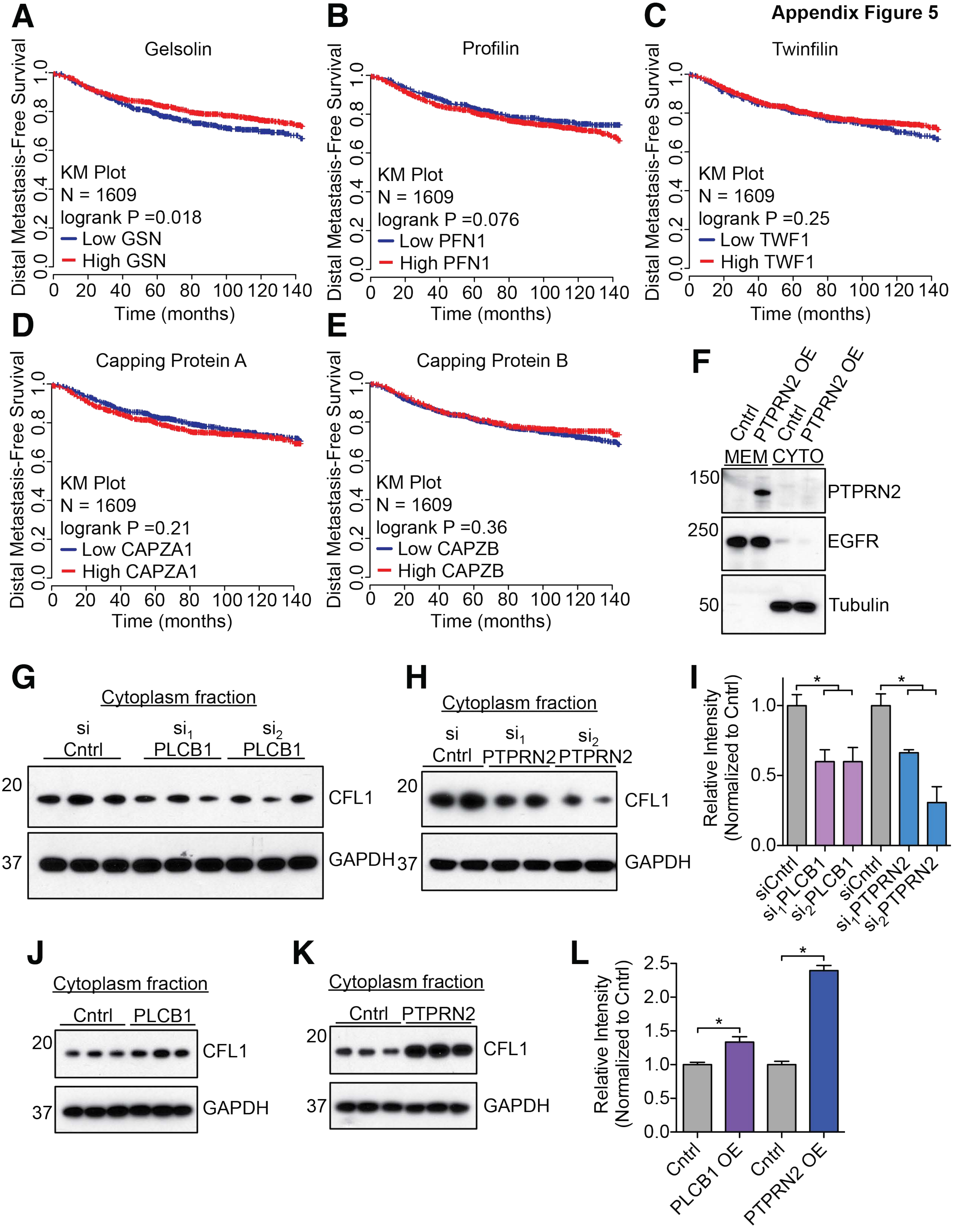

Appendix Figure 6

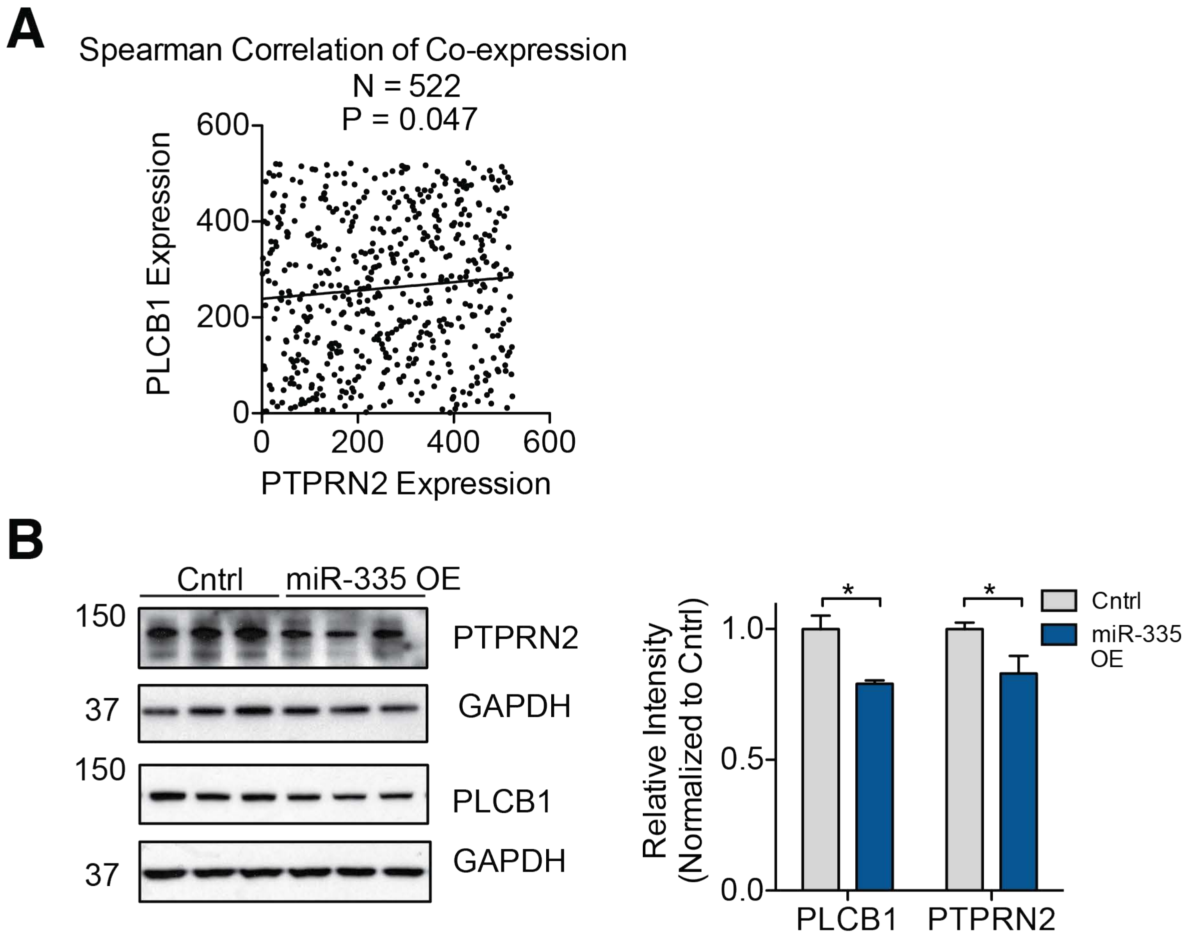

Appendix Figure Legends

Appendix Figure 1: PLCβ1 and PTPRN2 promote breast cancer metastasis in multiple breast cancer cell lines.

(**A**) Western blot analysis of LM2 cells transduced with shRNA targeting PTPRN2 or a control hairpin using anti-PTPRN2. GAPDH was used as a loading control. Densitometry analysis below blot is adjusted for GAPDH levels and normalized to shCntrl.

(**B**) LM2 cells were transfected with siRNA targeting PLCβ1 or a control siRNA. *PLCB1* expression levels were determined by qRT-PCR.

(**C**) Bioluminescence imaging quantification of lung colonization by 40,000 CNLM1a breast cancer cells transduced with shRNAs targeting PTPRN2 or a control hairpin. For shCntrl and sh_1PTPRN2, N = 6 mice/group. For sh_2PTPRN2, N = 5 mice. Right, H&E staining of representative lung sections.

(**D**) Bioluminescence imaging quantification of lung colonization by 40,000 CNLM1a breast cancer cells transfected with siRNAs targeting PLCβ1 or a control siRNA. N = 5 mice/group. Right, H&E staining of representative lung sections.

Error bars represent S.E.M.

Appendix Figure 2: PLCβ1 and PTPRN2 are required for migration and invasion in multiple breast cancer cell lines.

(**A-C**) LM2 cells transfected with siRNA targeting PTPRN2, PLCβ1, or a control siRNA were analyzed for viability (**A**), cytotoxicity (**B**), and caspase 3/7 activity (**C**) using the ApoTox-Glo Triplex assay (Promega). Caspase signal was normalized to siCntrl values for each group. N = 5 wells/group with 10,0000 cells per well. NS = not statistically significant.

(**D**) 100,000 LM2 cells transfected with siRNA targeting PLCβ1, PTPRN2, both PLCβ1 and PTPRN2, or a control vector were subjected to the migration assay.

(**E**,**F**) Migration assay by 100,000 BT-549 cells transfected with siRNA targeting PTPRN2 (**E**), PLCβ1 (**F**), or control siRNA. Data normalized to control values. N = 5 inserts/group.

(**G**,**H**) Migration assay by 100,000 CNLM1a cells transfected with siRNA targeting PTPRN2 (**G**), PLCβ1 (**H**), or control siRNA. Data normalized to control values. N = 5 inserts/group.

(**I**,**J**) Migration assay by 100,000 HCC-1806 cells transfected with siRNA targeting PTPRN2 (**I**), PLCβ1 (**J**), or control siRNA. Data normalized to control values. N = 5 inserts/group.

(**K**,**L**) Migration assay by 100,000 MDA-MB-468 cells transfected with siRNA targeting PTPRN2 (**K**), PLCβ1 (**L**), or control siRNA. Data normalized to control values. N = 5 inserts/group.

(**M**) 100,000 MDA-MB-231 cells overexpressing PLCβ1, PTPRN2, both PLCβ1 and PTPRN2, or a control vector were subjected to the migration assay.

Error bars represent S.E.M.

Appendix Figure 3: PLCβ1 and PTPRN2 abundance changes membrane PI(4,5)P2 levels in multiple breast cancer cell lines.

(**A**) Bioluminescence imaging quantification of lung colonization 1 day after injection of 40,000 LM2 cells treated with carrier incubated with PI(4,5)P2 or carrier alone. N = 6 mice/group.

(**B**) ELISA quantification of PI(4,5)P2 from membrane fractions of LM2 cells transfected with siRNA targeting PLCβ1, PTPRN2, or a control siRNA and MDA-MB-231 cells overexpressing PLCβ1, PTPRN2, or a control vector. N = 5 wells/group.

(**C**, **D**) Mean fluorescence intensity of membrane PI(4,5)P2 was analyzed in MDA-MB-468 (**C**) and CNLM1a1 (**D**) cells transfected with siRNA targeting PLCβ1, PTPRN2, or a control siRNA and immunostained with PI(4,5)P2 antibody (red) and DAPI (blue) using fluorescence microscopy. Left, representative images. N = 30 cells/group. Scale bar, 50 μm.

Error bars represent S.E.M.

Appendix Figure 4: PI(4,5)P2 abundance governs migration and invasive capacity.

(**A**) MDA-MB-231 cells transfected with Lyn_{11}-FRB and INPP5E-FKBP, treated with either DMSO or 10 nm rapamycin, and immunostained with anti-PI(4,5)P2 antibody (green) and DAPI (blue). Left, quantification of membrane PI(4,5)P2 mean fluorescence intensity. Right, representative images. N = 50 cells/group. Scale bar, 10 µm.

(**B**) MDA-MB-231 cells overexpressing PTPRN2 or a control vector were treated with carrier incubated with PI(4,5)P2, carrier incubated with PI4P, or carrier alone, and subjected to the migration assay. N = 5 inserts/group.

(**C**, **D**) Western blot analysis of MDA-MB-231, LM2, CN34, and CNLM1a1 cells using anti-PIP5K1A, anti-PIP5K1C (**C**), anti-PIP5K1B (**D**), and anti-GAPDH. Densitometry values below blots are adjusted for GAPDH levels and normalized to MDA levels.

(**E**) Western blot analysis of LM2 cells overexpressing PIP5K1A or a control vector using anti-PIP5K1A. GAPDH was used as a loading control. Densitometry values below blot are adjusted for GAPDH levels and normalized to Cntrl value.

(**F**) Mean fluorescence intensity of membrane PI(4,5)P2 was analyzed in LM2 cells overexpressing PIP5K1A or a control vector immunostained with PI(4,5)P2 antibody (red) and DAPI (blue) and analyzed using fluorescence microscopy. N = 50 cells/group. Scale bar, 50 µm. Error bars represent S.E.M.

Appendix Figure 5: PLCβ1 and PTPRN2 regulate localization of cofilin, a PI(4,5)P2-binding protein.

(**A**-**E**) Kaplan-Meier curves representing distal metastasis-free survival of a cohort of breast cancer patients (N = 1609) as a function of their primary tumor's indicated gene expression levels (Data from KMPlot(Gyorffy et al., 2010)). Patients' primary tumors' indicated gene expression levels were classified as low (blue) or high (red) expression. *GSN* (**A**), *PFN1* (**B**), *TWF1* (**C**), *CAPZA1* (**D**), *CAPZB* (**E**).

(**F**) Membrane and membrane-associated proteins (MEM) and cytoplasmic proteins (CYTO) were collected from MDA-MB-231 cells overexpressing PTPRN2 or a control vector. Protein fractions were subjected to Western blot analysis for PTPRN2, EGFR, and Tubulin.

(**G**, **H**) Western blot analysis of cytoplasmic fractions from LM2 cells with knockdown of PLCβ1 (**G**) or PTPRN2 (**H**) using anti-CFL1 and anti-GAPDH.

(**I**) Densitometry analysis of western blots in (**G**, **H**).

(**J**, **K**) Western blot analysis of cytoplasmic fractions from MDA-MB-231 cells overexpressing PLCβ1 (**J**), PTPRN2 (**K**), or a control vector using anti-CFL1 and anti-GAPDH.

(**L**) Densitometry analysis of western blots in (**J**, **K**).

Error bars represent S.E.M.

Appendix Figure 6: PTPRN2 and PLCβ1 are negatively regulated by the metastasis suppressor microRNA miR-335.

(**A**) Spearman correlation of co-expression of *PTPRN2* and *PLCB1* in the primary tumors of a cohort of breast cancer patients (N = 528). Data from the TCGA Research Network (Cancer Genome Atlas, 2012).

(**B**) Western blot analysis of PTPRN2 and PLCβ1 in whole cell lysate from LM2 cells over-expressing miR-335 or a control vector. Right, densitometry quantification of Western blots normalized to GAPDH.

Appendix Table 1: Cloning primers

PTPRN2<br>Vector: pBABE-puro	F: CCGGCCGAATTCGGGCCGCCGCTCCCGCTGCTG
	R: CCGGCCCTCGAGTCACTGGGGAAGGGCCTTGAG
PLCB1<br>Vector: pBABE-puro	F: CCGGCCTACGTAATGGCCGGGGCTCAACCCGGA
	R: CCGGCCGTCGACTCACAGAGGAGTATCAAATTC
CFL1-Lck<br>Vector: pEGFP-N1	F:CCGGCCGAATTCATGGGATGCGTCTGCTCAAGCGCCTCCGGTGTGGCTGTC
	R:CCGGCCGGATCCCGGGATAACAAAGGCTTGCCCTCCAGGGAG
PIP5K1A<br>C-terminal FLAG<br>Vector: pBABE-puro	F: CCGGCCTACGTAATGGCGTCGGCCTCCTCCGGG
	R:CCGGCCGTCGACTTACTTGTCGTCATCGTCTTTGTAGTCATGGGTGAACTCTGACTCTGC

Appendix Table 2: shRNA sequences

Control	CCGGCAACAAGATGAAGAGCACCAACTCGAGTTGGTGCTCTTCATCTTGTTGTTTTT
PTPRN2 #1	CCGGAGGTGCTAAAGAGATTGATATCTCGAGATATCAATCTCTTTAGCACCTTTTTT
PTPRN2 #2	CCGGCGACGATGATAGACTTTACCACTCGAGTGGTAAAGTCTATCATCGTCGTTTTT

Appendix Table 3: Mutagenesis primers

PTPRN2 C945A	F:GCCGTTCTTGTCCAATAATTGTTCATGCCAGTGACGGTGCAG
	R:CTGCACCGTCACTGGCATGAACAATTATTGGACAAGAACGGC
PLCB1 H331Q	F: ATTTCATTAATTCCTCGCAAAACACCTACCTCACAGCTG
	R: CAGCTGTGAGGTAGGTGTTTTGCGAGGAATTAATGAAAT

Appendix Table 4: siRNA sequences

Control	CGUUAUCGCGUAUAUACGCGAU
PTPRN2 #1	CCUACUGAGCGGACAGAAAGAAGCC
PTPRN2 #2	GGCAUUGAGCAAGCUAUGAGGGUCC
PLCB1 #1	CGCUAAGAAAUAAUUGAUGGAGCCA
PLCB1 #2	GGCGUUAAUCAUAAAGAAGCAAGAA
CFL1 #1	GCAAGCAAACUGCUACGAGGAGGUC
CFL1 #2	ACGACAUGAAGGUGCGUAAGUCUUC
CFL1 #3 (3'UTR targeting)	CUCAUGGAAGCAGGACCAGUAAGGG

Appendix Table 5: Quantitative PCR primers

PTPRN2	F: GGCCAAAGGTGCTAAAGAGA
	R: TGTCAGCGCGAACTCAAA
PLCB1	F:TCTGGAATGCAGGTTGTCAG
	R:GCCACTCTTCCCGTTGTATT
CFL1	F: TGTCAAGATGTGCCAGATAA
	R: GCCCAGAAGATAAACACCAGAT
PIP5K1A	F: CGGCCCGATGATTACTTGTAT
	R: CGTCGCTGGACACATAGAATAG

APPENDIX SUPPLEMENTARY METHODS

Cellular viability, cytotoxicity, and caspase activity

Cellular viability, cyototoxicty, and caspase 3/7 activity were measured using the ApoTox-Glo Triplex Assay (Promega) according to the manufacturer's instructions. 10,000 cells were seeded per well. Caspase 3/7 luminescence activity was normalized to viability signal.

PI(4,5)P2 Mass ELISA

PI(4,5)P2 was extracted from breast cancer cells and quantified using a PI(4,5)P2 Mass ELISA (Echelon Biosciences) according to the manufacturer's instructions. To extract PI(4,5)P2, cell or membrane pellet was incubated on ice for 5 minutes with 1 mL ice cold 0.5 M TCA. Samples were centrifuged at 1,000 *g* for 7 minutes at 4°C. The resulting pellet was washed with 1 mL 5% TCA/1 mM EDTA twice. To extract neutral lipids, 1 mL MeOH:$CHCl_3$ (2:1) was added, the samples were vortexed for 10 minutes, and centrifuged at 1,000 *g* for 5 minutes. Neutral lipid extraction was performed twice. To extract acidic lipids, 750 µL MeOH: $CHCl_3$:12 N HCl (80:40:1) was added and samples were vortexed for 25 minutes followed by centrifugation for 1,000 *g* for 5 minutes. 250 µL of $CHCl_3$ and 450 µL of 0.1 N HCl was added to the supernatant. The sample was vortexed for 30 seconds and centrifuged at 1,000 *g* for 5 minutes to separate organic and aqueous phases. The organic phase was collected and dried under argon. Dried lipids were stored at -20°C and reconstituted in assay buffer immediately prior to ELISA procedure.

Membrane fractionation was performed as previously described (van Rheenen et al., 2007). Briefly, cells were lysed in ice-cold lysis buffer (50 mM Tris, pH 7.5, 300 mM NaCl, 5 mM EGTA, 20 mM DTT, 1% Triton X-100). A fraction of the lysate was measured for protein content by BCA assay (Thermo) to normalize cell number. The harvested cell lysate was mixed with 60%

OptiPrep (Sigma) to yield 40% OptiPrep final. The lysate containing 40% OptiPrep was transferred to the bottom of a centrifuge tube. 1 ml 30% OptiPrep was added on top of 40% OptiPrep lysate. 300 µl of 5% OptiPrep was added on top of 30% OptiPrep. Tubes were centrifuged for 12h at 4°C at 100,000 *g.* Gradients were collected, and low-density membrane (top) fractions were used in further analysis.

APPENDIX REFERENCES

Cancer Genome Atlas N (2012) Comprehensive molecular portraits of human breast tumours. Nature 490: 61-70

Gyorffy B, Lanczky A, Eklund AC, Denkert C, Budczies J, Li Q, Szallasi Z (2010) An online survival analysis tool to rapidly assess the effect of 22,277 genes on breast cancer prognosis using microarray data of 1,809 patients. Breast Cancer Res Treat 123: 725-31

van Rheenen J, Song X, van Roosmalen W, Cammer M, Chen X, Desmarais V, Yip SC, Backer JM, Eddy RJ, Condeelis JS (2007) EGF-induced PIP2 hydrolysis releases and activates cofilin locally in carcinoma cells. J Cell Biol 179: 1247-59
