# Supplementary material for: PTPRN2 and PLCβ1 promote metastatic breast cancer cell migration through PI(4,5)P2‐dependent actin remodeling
Source: EMBO J. 2015 Nov 30;35(1):62–76. doi: 10.15252/embj.201591973 (PMC4717998; doi:10.15252/embj.201591973)
Supplement: Supplementary file 2 — Expanded View Figures PDF [file EMBJ-35-062-s002.pdf]

Expanded View Figures

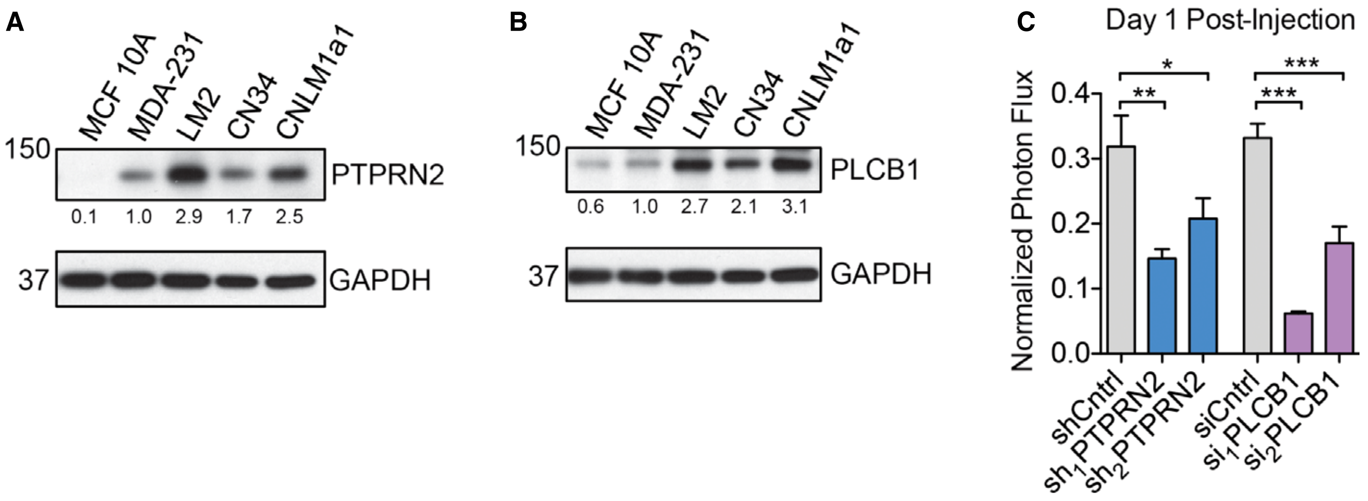

Figure EV1. PTPRN2 and PLCβ1 promote breast cancer metastasis.

A, B Western blot analysis of MCF 10A, MDA-MB-231, LM2, CN34, and CNLM1a1 cell lysate using anti-PTPRN2 (A) or anti-PLCβ1 (B). GAPDH was used as a loading control. Densitometry analysis below the blots is adjusted for GAPDH levels and normalized to MDA-MB-231 values.

C Bioluminescence imaging quantification of lung colonization 1 day after injection of 40,000 LM2 cells with knockdown of PTPRN2, PLCβ1, or control cells. For shCntrl, sh_1PTPRN2: $N = 5$ mice/group. For sh_2PTPRN2: $N = 6$ mice. For siCntrl: $N = 5$ mice. For si_1PLCb1, si_2PLCb1: $N = 6$ mice/group. Error bars represent SEM. $^*P < 0.05$, $^{**}P < 0.01$, $^{***}P < 0.001$.

 © 2015 The Authors

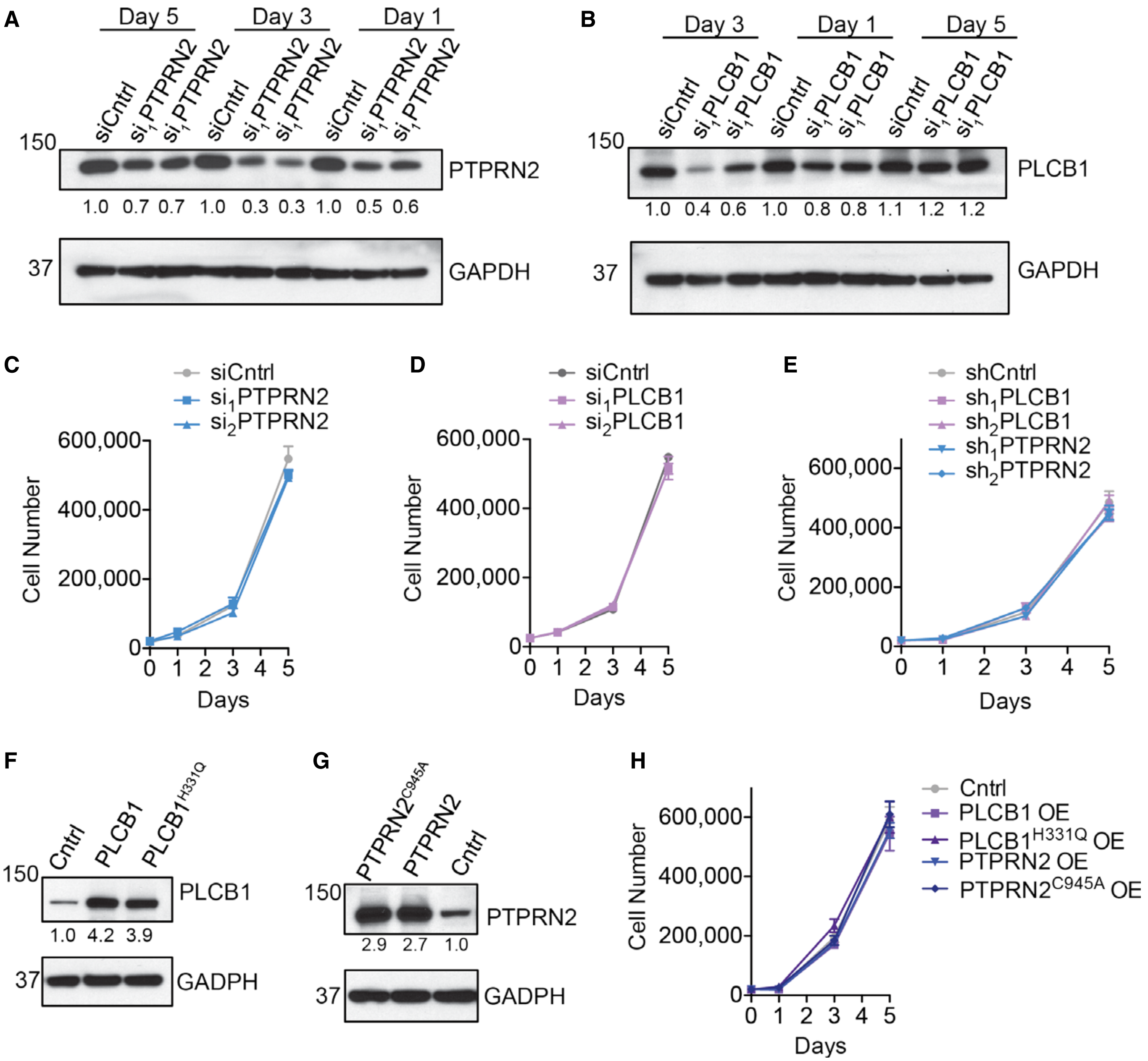

Figure EV2. PLCβ1 and PTPRN2 drive metastatic migration and invasion.

A, B Western blot analysis of LM2 cells transfected with siRNA targeting PTPRN2, PLCβ1, or a control siRNA using anti-PTPRN2 (A) and anti-PLCβ1 (B) at various time points post-transfection. GAPDH was used as a loading control. Densitometry analysis below the blots is adjusted for GAPDH levels and normalized to siCntrl values on day 1.

C, D Proliferation of 20,000 LM2 cells transfected with siRNA targeting PTPRN2 (C), PLCβ1 (D), or a control siRNA. N = 3 wells/group.

E Proliferation of 20,000 LM2 cells transduced with hairpins targeting PLCβ1, PTPRN2, or a control hairpin. N = 3 wells/group.

F, G Western blot analysis of MDA-MB-231 cells overexpressing PLCβ1, PLCβ1^{H331Q} (F), PTPRN2, PTPRN2^{C945A} (G), or a control vector using anti-PTPRN2 (F) or anti-PLCβ1 (G). GAPDH was used as a loading control. Densitometry analysis below the blots is adjusted for GAPDH levels and normalized to Cntrl values.

H Proliferation of 20,000 MDA-MB-231 cells overexpressing PLCβ1, PLCβ1^{H331Q}, PTPRN2, PTPRN2^{C945A}, or a control vector. N = 3 wells/group.

Data information: Error bars represent SEM.

© 2015 The Authors

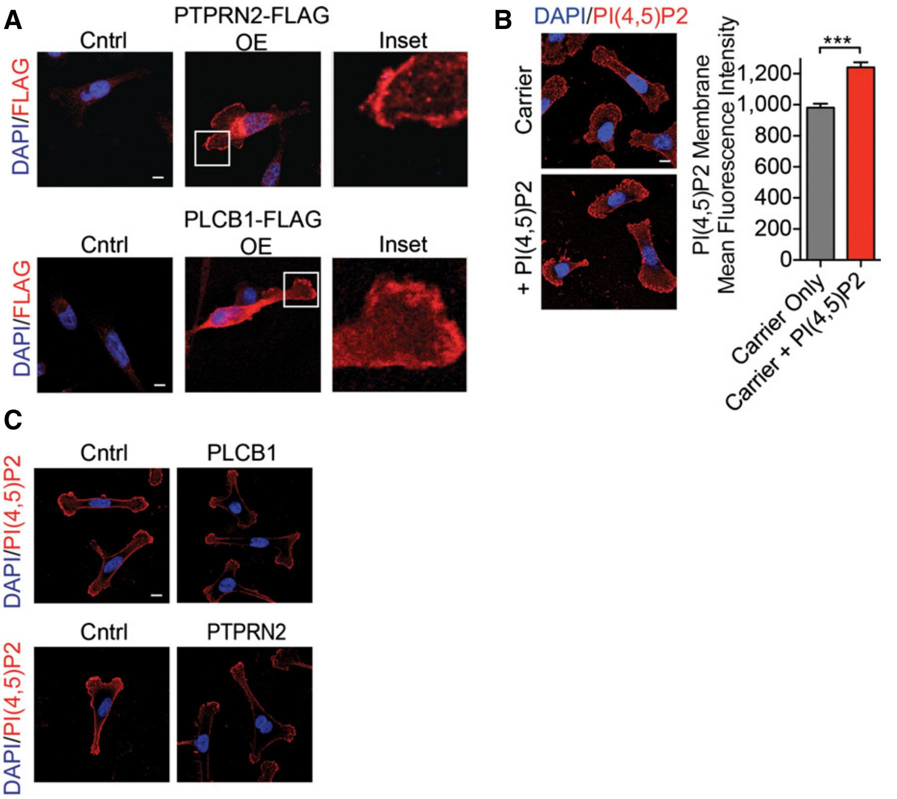

Figure EV3. PLCβ1 and PTPRN2 regulate membrane PI(4,5)P_2 levels.

A Representative images of MDA-MB-231 cells retrovirally transduced with PTPRN2-FLAG, PLCβ1-FLAG, or control vector and immunostained with anti-FLAG (red) and DAPI (blue). Scale bar, 10 μm.

B Mean fluorescence intensity of membrane PI(4,5)P_2 was analyzed in LM2 cells treated with carrier incubated with PI(4,5)P_2 or carrier alone. Cells were immunostained with anti-PI(4,5)P_2 antibody (red) and DAPI (blue) and analyzed using fluorescence microscopy. $N = 50$ cells/group. Scale bar, 10 μm. Error bars represent SEM. ***$P < 0.001$.

C Representative fluorescence images of MDA-MB-231 overexpressing PTPRN2, PLCβ1, or control vector immunostained with anti-PI(4,5)P_2 antibody (red) and DAPI (blue). Scale bar, 10 μm.

◀ **Figure EV4. PLCβ1 and PTPRN2 facilitate cofilin localization and activity.**

A Western blot analysis of whole-cell lysate of LM2 cells transfected with a control siRNA or siRNAs targeting PTPRN2 or PLCβ1 using anti-CFL1, anti-β-actin, and anti-GAPDH.

B Western blot analysis of whole-cell lysate of MDA-MB-231 cells overexpressing PTPRN2, PLCβ1, or a control vector using anti-CFL1, anti-β-actin, and anti-GAPDH.

C LM2 cells transfected with siRNA targeting PTPRN2, PLCβ1, or a control siRNA were immunostained for CFL1 (red) and DAPI (blue). Left, quantification of membrane mean fluorescence intensity of CFL1. Right, representative images. $N = 50$ cells/group. Scale bar, 10 μm. Error bars represent SEM. ***$P < 0.001$.

D MDA-MB-231 cells overexpressing PTPRN2, PLCβ1, or a control vector were immunostained for CFL1 (red) and DAPI (blue). Left, quantification of membrane mean fluorescence intensity of CFL1. Right, representative images. $N = 50$ cells/group. Scale bar, 10 μm. Error bars represent SEM. ***$P < 0.001$.

E Representative images for Fig 5F. LM2 cells were transfected with siRNA targeting PLβ1 or a control siRNA and subjected to the barbed end assay. Cells were stained for biotin–actin (red), DAPI (blue). Scale bar, 20 μm.

 © 2015 The Authors

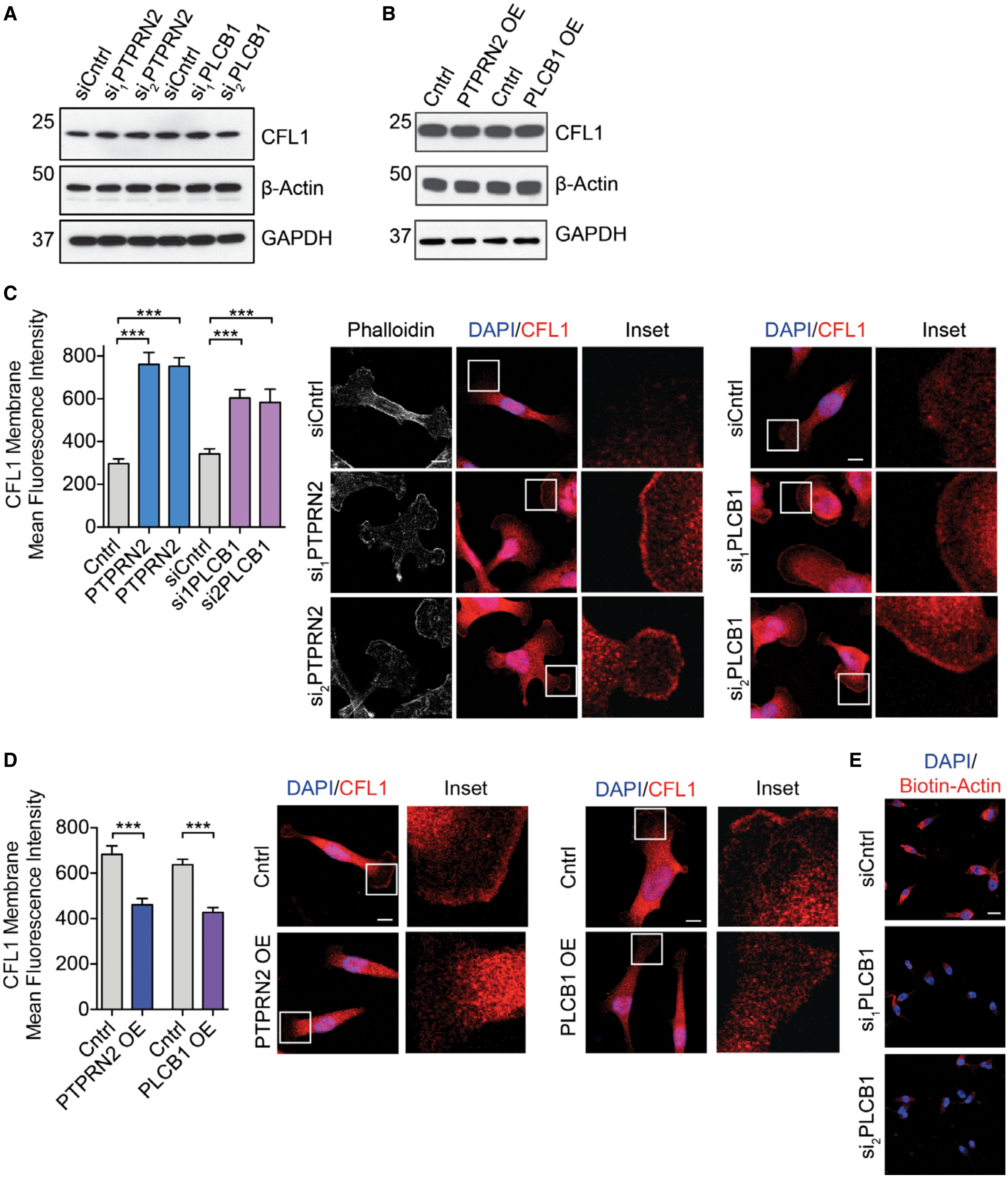
A
siCntrl
si_1PTPRN2
si_2PTPRN2
siCntrl
si_1PLCB1
si_2PLCB1
25
CFL1
50
β-Actin
37
GAPDH
B
Cntrl
PTPRN2 OE
Cntrl
PLCB1 OE
25
CFL1
50
β-Actin
37
GAPDH
C
CFL1 Membrane Mean Fluorescence Intensity
***
***
***
***
800
600
400
200
0
Cntrl
PTPRN2
PTPRN2
siCntrl
si1PLCB1
si2PLCB1
Phalloidin
DAPI/CFL1
Inset
siCntrl
si_1PTPRN2
si_2PTPRN2
si_1PLCB1
si_2PLCB1
D
CFL1 Membrane Mean Fluorescence Intensity
***
***
800
600
400
200
0
Cntrl
PTPRN2 OE
Cntrl
PLCB1 OE
Inset
Cntrl
PTPRN2 OE
PLCB1 OE
E
DAPI/
Biotin-Actin
siCntrl
si_1PLCB1
si_2PLCB1

Figure EV4.

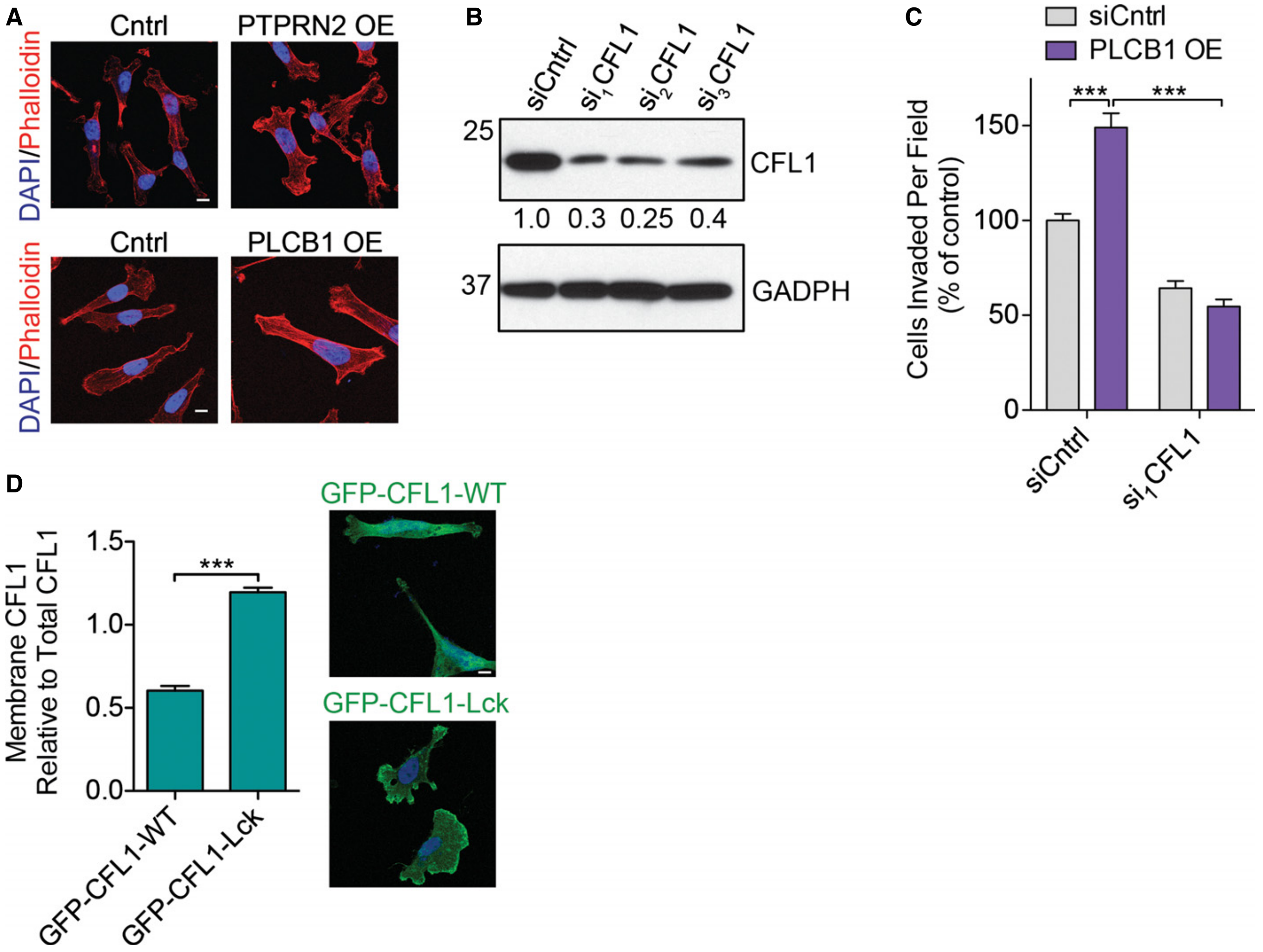

Figure EV5. PLCβ1 and PTPRN2 act upstream of cofilin-mediated actin dynamics.

A Representative images for Fig 6B. MDA-MB-231 overexpressing PTPRN2, PLCβ1, or control vector were stained with phalloidin (red) and DAPI (blue) and analyzed using fluorescence microscopy. Scale bar, 10 μm.

B Western blot analysis of MDA-MB-231 cells transfected with siRNA targeting CFL1 or a control siRNA using anti-CFL1. GAPDH was used as a loading control. Densitometry values below the blot are adjusted for GAPDH levels and normalized to siCntrl value.

C MDA-MB-231 cells were transfected with siRNAs targeting CFL1 or a control siRNA in the setting of control or PLCβ1 overexpression and subjected to the invasion assay. $N = 5$ inserts/group. Error bars represent SEM. ***$P < 0.001$.

D MDA-MB-231 were transfected with siRNA targeting the 3′ UTR of *CFL1* to deplete endogenous CFL1 and further transfected with plasmids encoding either GFP-CFL1-WT or GFP-CFL1-Lck (green) and immunostained with DAPI (blue). Left, quantification of membrane mean fluorescence intensity of GFP-CFL1 as analyzed by fluorescence microscopy. Right, representative images. $N = 50$ cells/group. Scale bar, 10 μm. Error bars represent SEM. ***$P < 0.001$.

© 2015 The Authors
